# Mature cystic extragonadal teratoma in Douglas’ pouch: Case report and literature review

**DOI:** 10.3389/fmed.2022.985235

**Published:** 2023-02-24

**Authors:** Yun Yang, Mengru Zhao, Mengyue Chen, Huimin Tang, Zhenyue Qin, Junling Liu, Huihui Wang, Mingyue Bao, Jiming Chen, Bairong Xia

**Affiliations:** ^1^Dalian Medical University Graduate School, Dalian, China; ^2^Department of Gynecology, The Affiliated Changzhou No. 2 People’s Hospital of Nanjing Medical University, Changzhou, China; ^3^Division of Life Sciences and Medicine, Department of Gynecology, The First Affiliated Hospital of USTC, University of Science and Technology of China, Hefei, China

**Keywords:** mature cystic teratoma, Douglas’ pouch, case report, old women, literature review

## Abstract

Teratomas often occur in the gonads, while Extragonadal mature cystic teratomas are reported occasionally, with the most common site being the omentum. Teratoma in the Douglas sac is extremely rare. we report a rare case of mature cystic Teratoma in the Douglas sac in a 71-year-old woman who underwent laparoscopic surgery. A cyst with a diameter of approximately 6 cm from Douglas was found during surgery, and the mass was separated from both ovaries. Microscopically, the cyst was a mature cystic teratoma that did not originate from the ovary.

## Introduction

Teratomas are germ cell-derived tumors with the potential to differentiate into somatic cells. Most teratomas contain at least two types of embryonic tissue components. According to differentiation degrees of the tissues contained, teratomas can be divided into mature ones and immature ones. Mature cystic ovarian teratoma, also known as dermoid cyst, is a benign tumor, accounting for 85–97% of germ cell tumors and more than 95% of teratomas. It is primarily composed of ectodermal, mesodermal, and endodermal mature tissues, and usually dominated by ectoderm, mostly cystic, which can be monocystic or polycystic. The content is oil-like, often mixed with hair, and the cyst wall is often lined with multi-layered squamous epithelium and appendage hair follicles, sebaceous glands, sweat glands, etc. Immature teratoma is a malignant germ cell tumor that reproduces the differentiation characteristics of embryos and fetal tissues. Its tissues contain different amounts of immature tissues, largely primitive and embryonic neuroectodermal tissues. Teratomas often occur in the gonads, while extragonadal mature cystic teratomas are occasionally reported, with the most common site being the omentum majus (1). However, teratoma in Douglas’ pouch is extremely rare and its exact etiology is unknown. Here we have a rare case of mature cystic teratoma in Douglas’ pouch in a 71-year-old woman who underwent laparoscopic surgery. A cyst with a diameter of approximately 6 cm in Douglas’ pouch was found during surgery, and the mass was separated from both ovaries. Microscopically, the cyst was a mature cystic teratoma.

## Case description

A 71-year-old women was admitted to the hospital because of “a prolapse of vaginal mass for more than 3 years and a pelvic mass found 1 month ago.” When the patient stood upright, a broad-bean-sized mass prolapsed out of the vagina, and the symptom disappeared by itself when the patient rested. She has been post-menopausal for 25 years, with 4 pregnancies and 3 children, and received cataract surgery 6 years ago. Physical examination showed no obvious mass palpated in the abdomen and no tenderness. Gynecological examination showed no abnormality in the vulva and vagina, atrophic and smooth cervix, uterus in the middle position and metratrophia, no tenderness, and a cystic mass of a diameter of 6 cm felt behind the uterus with clear boundary, smooth surface, and acceptable mobility without tenderness. No obvious mass was palpated in bilateral adnexal areas. Tumor markers are largely normal. Pelvic ultrasound showed a mixed echogenic mass on the left side of the pelvis. The mass size was about 7.4*5.4 cm.

**FIGURE 1:**
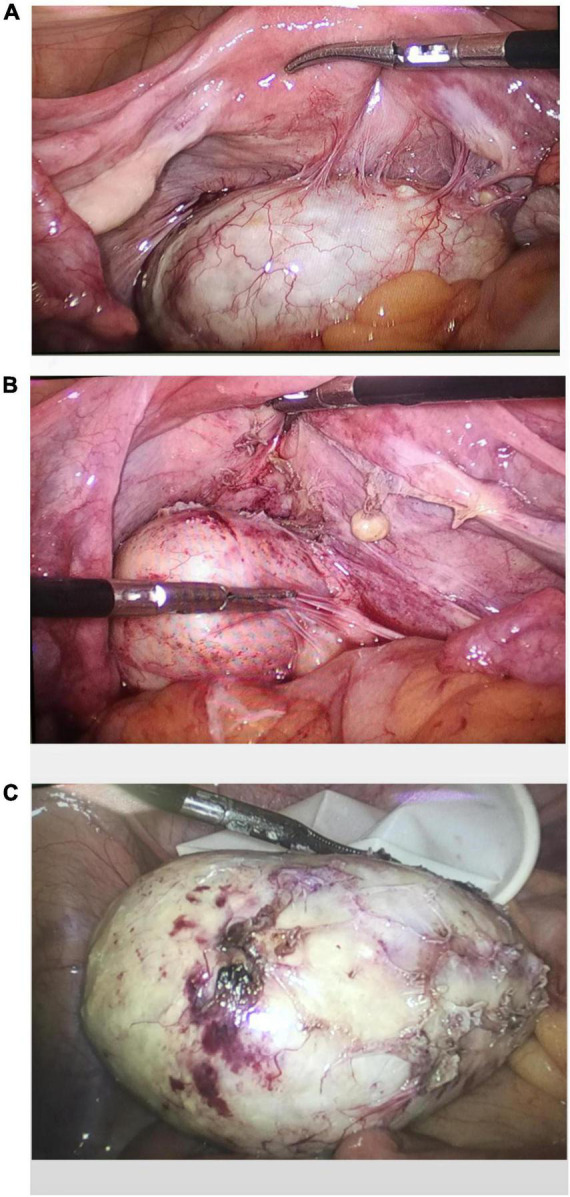
Intraoperative exploration revealed a teratoma in the utero-rectal fossa, adherent to the posterior uterine wall, right adnexa, and mesentery **(A)**. The teratoma was disconnected from the posterior uterine wall, right adnexa, and mesentery **(B)**. Gross observation of the specimen **(C)**.

**FIGURE 2:**
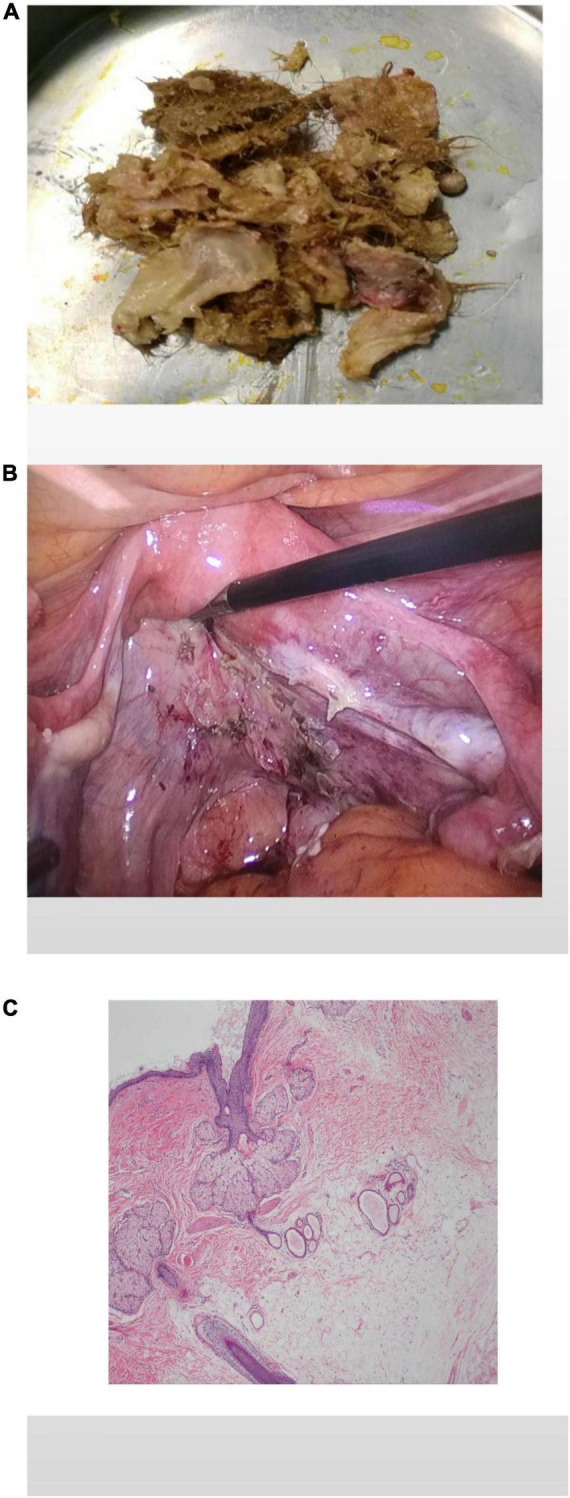
The specimen contains hair, bone, and tooth-like tissues **(A)**. The pelvis and bilateral adnexal areas after complete resection of the teratoma showed no obvious abnormality **(B)**. Histological pictures **(C)**.

A laparoscopic exploratory operation was performed under general anesthesia on 10 August 2017. During the operation, it could be observed that the uterine surface was smooth, and a mass with a diameter of about 6 cm was seen in Douglas’ pouch. The surface of the mass was smooth, without obvious connection with the uterus and bilateral appendages, but its back was tightly adhered to the posterior wall of the uterus, the right appendages, and the mesentery (Figure 1A). The bilateral ovarian atrophied, being grayish-white and with an intact surface. We used scissors to separate the adhesion between the mass and its surrounding tissues (Figure 1B) until the mass was completely isolated (Figure 1C). Then, the specimen was put into a specimen bag made of a sterile glove. Yellow liquid flew out after the mass was cut and contained hair, bone, and tooth-like tissues (Figure 2A). Both ovaries were regarded normal considering the patient’s age (Figure 2B).

The patient had an uneventful postoperative course. Pathological examination after surgery showed that the tumor was mature cystic teratoma (Figure 2C).

## Discussion

Mature cystic teratoma is a congenital tumor that originates from pluripotent stem cells and contains tissues from at least two of the three germ cell layers. They usually have a parthenogenetic origin with a 46, XX karyotype and characteristic stagnation after Meiosis I. Meckel (2) first described extragonadal teratomas in 1815. The most common location was the omentum, followed by Douglas’ pouch, liver, and diaphragmatic inguinal canal. In addition, teratomas may also be found in the reproductive tract of the uterus, cervix, and oviduct.

There are three proposed theories about the causes of these extragonadal sites: (1) Primary teratoma may originate from displaced germ cells. (2) Teratoma may occur in the supernumerary ovary. (3) Teratoma may be caused by automatic amputation of ovarian dermoid cyst. The first mechanism is during early fetal development when germ cells migrate from the yolk sac along the hindgut to the reproductive crest. Germ cells become arrested between the yolk sac entoderm and the dorsal mesentery (3). The second mechanism is teratoma caused by multiple or accessory ovaries. It was first described by Wharton (4). Supernumerary ovaries are completely separated from normal ovaries and come from single primordial ones. Accessory ovaries refer to the multiple ovarian tissues found in near-normal ovaries, which may be connected to or developed from normal ovaries. In embryology, the migration of primitive cells from the yolk sac to a gonadal ridge and delayed germ cell migration may be the cause for the formation of polycystic ovaries. The third mechanism was first described by Thornton. In subacute or chronic torsion, teratomas may adhere to adjacent structures and form new collateral circulation. In rare cases, the tumors may be completely detached from their pedicle, leading to parasitic dermoid cysts (5). Auto amputation is considered the most common cause of omentum EGT and Douglas’ pouch and sacral ligament EGT, and 70% of upper abdominal EGT may be caused by the displacement of primordial germ cells (6). It seemed to be more frequently seen on the right side, which is likely due to the colon sigmoideum preventing left-sided torsion (7). In our case, the possibility of ovarian auto amputation could be ruled out for the time being, because the bilateral ovarian morphology was intact and the mechanism needed further exploration.

The cases in many reports are parasitic dermoid cysts in the omentum majus, while the cases of teratomas located in the Douglas’ pouch are rare. We focused on the parasitic cysts located in the Douglas’ pouch to investigate the disease characteristics as reference for clinical diagnosis and treatment. We performed a PubMed search using keywords “extragonadal” or “parasitic,” “teratoma,” “Douglas,” or “cul-de-sac,” and found 24 articles. The clinical data of the 24 cases of parasitic dermoid cysts in Douglas’ pouch is given in Table 1 (7–29). The patient age ranged from 23 to 83, and 11 patients had abdominal pain as the primary symptom and the other patients experienced no abdominal pain. One of the patients had no obvious clinical symptoms, and Douglas’ pouch teratoma was found during cesarean section at 39 weeks of gestation. Another patient found the teratoma by physical examination at 39 days of gestation. Three of 24 patients had teratoma in the uterosacral ligament. The maximum size of the tumor was 15 cm × 15 cm × 10 cm. Nine of 24 patients had normal levels of tumor markers (CA-125, CA-19-9, alpha fetoprotein, and carcinoembryonic antigen), four had elevated levels of tumor markers, with CA 19-9 up to 631.23 U/mL, and CA125 up to 110.5 U/mL. A total of 12 of 24 cases were considered to be a result of auto amputation, 3 were considered a result of displaced primordial germ cells, and the causes of the remaining cases were unknown.

**TABLE 1:** Case summary of extragonadal teratoma in Douglas’ pouch.

References	Age	Symptom	Pregnancy history	Operation history	Size (cm)	Tumor marker	State of ovaries	Etiology
Lefkowitch et al. (8)	40	Dysuric	G0P0	None	11 × 9 × 7	Not described	Normal	Reproductive cell migration during the germ cell cycle
Turhan et al. (9)	30	Yellow-green vaginal secretions	G3P2	Not described	5 × 4 × 3	Not described	Normal	Unknown
Chen et al. (10)	61	Pain in the abdomen	G6P3, menopausal	Left ovarian cyst resection	3 × 3	Normal	Right ovarian cyst 4 cm × 3 cm, left ovarian atrophy	Possibility of spillage during previous cystectomy leading to recurrence
Takeda et al. (11)	49	Asymptomatic	G4P3	Not described	4, uterosacral ligament	Normal	Normal	Unknown
Kobayashi et al. (12)	61	Asymptomatic	G4P2	Not described	Head size	Normal	Normal	Auto-amputation
Kusaka and Mikuni (7)	24	Pain in the abdomen	G0P0	Not described	5	Normal	Absence of left ovary, normal right ovary	Auto-amputation
Khoo et al. (13)	29	Pain in the abdomen	Not described	None	7	Normal	Right ovarian cyst 7 cm × 7 cm	Auto-amputation
Moawad et al. (14)	59	None	Not described	Renal cell tumor	2 × 4, uterosacral ligament	Not described	The left ovary is absent, and the right ovarian teratoma is about 10 cm in size	Unknown
Bartlett et al. (15)	29	Pain in the abdomen	Not described	Not described	6 × 6	CA-125 38 U/mL	The left ovary and uterus are normal. The right ovary is shrunk and the fallopian tube is dilated	Auto-amputation
Peitsidou et al. (16)	33	None	G1P0	Not described	9	Not described	The right ovary is missing, the left side and uterus are normal	Auto-amputation
Sinha et al. (17)	23	Pain in the abdomen	P0	History of right ovarian teratoma resection	4 × 3	Normal	Left ovary normal	Auto-amputation
Matsushita et al. (18)	69	Pain in the abdomen	Not described	Not described	7	CA125 was 110.5 U/m, CA19-9 was 92 U/mL	Right ovarian atrophy	Auto-amputation
Bambao and Liu (19)	50	Asymptomatic	Not described	Not described	Not described	Not described	Bilateral ovarian atrophy	Unknown
Tokunaga et al. (20)	37	None	P0	Postoperative chemotherapy for left ovarian immature teratoma	Not described	CA19-9 was 94.1 U/mL	Absence of left ovary, right ovarian cyst 6 cm	Unknown
Takeda et al. (11)	26	Asymptomatic with 39 days of pregnancy	G1P0	Not described	2.9	CA 19-9 was 631.23 U/mL	Left ovarian cyst 3 cm	Unknown
Eda et al. (21)	83	Autopsy	Not described	Not described	8 × 7 × 6	Not described	Absence of left ovary, right ovarian normal	Unknown
Koo et al. (22)	34	Asymptomatic	G3P2	Not described	4 × 3, uterosacral ligament	Normal	Right ovarian normal	Unknown
Makni et al. (23)	55	Pain in the abdomen	Not described	Not described	2 × 2	Not described	Normal	Auto-amputation
Ohshima et al. (24)	20	Pain in the abdomen	None	Not described	7 × 6 × 2		Normal	Reproductive cell migration during the germ cell cycle
Kakuda et al. (25)	41	Pain in the abdomen	Not described	None	4	Not described	Absence of left ovary, right ovarian normal	Auto-amputation
John (26)	32	Asymptomatic	P2	Not described	6	Not described	Left ovary and left fallopian tube missing, right appendage normal	Auto-amputation
Kim et al. (27)	34	Pain in the abdomen	G0P0	Not described	5 × 3	(CA) 125; 10.4 U/mL, CA 19-9; 2 U/mL	Absence of left ovary, right ovarian normal	Auto-amputation
Sethi and Purkait (28)	40	Pain in the abdomen	Not described	Not described	15 × 15 × 10	CA-125 was 11.7 U/mL CA 19-9 was 35.26 U/mL	Normal	Reproductive cell migration during the germ cell cycle
Daccache et al. (29)	42	None	G3P3	None	2	Not described	Right ovarian cystadenoma 8 cm	Auto-amputation
Present case	71	Prolapse of vaginal mass	G4P3, menopausal	Operated for cataracts 6 years ago	7.4 × 5.4	None	Bilateral ovarian atrophy	Unknown

When extragonadal teratomas develop in the uterus, cervix, and oviduct, symptoms such as abnormal uterine bleeding and vaginal masses may occur. Patients with Douglas’ pouch teratomas may suffer from hypo gastralgia (30). Some patients may have no clinical symptoms until their teratomas were found during physical examinations. In this case, the abdominal pain was not obvious, and the patient was in menopause, with prolapse of vaginal mass and less vaginal secretion as the main manifestations. Preoperative diagnosis of this disease is highly difficult, and transvaginal ultrasound is the preferred method of examination. MRI or CT scan of the pelvis can help with preoperative diagnosis. CT images often show the presence of fat and calcification. The current data shows that tumor markers have little effect on the diagnosis of the disease. Intraoperative and postoperative pathological analysis is the main evidence to establish the diagnosis. Although not all cases have accurate histopathological diagnosis, the mass is usually composed of necrotic and calcified tissues, and residual ovarian components can be found in a small number of cases (15). The preferred method for the treatment of this disease is surgery. For benign teratomas, laparoscopic cystectomy can be the preferred surgical method, and the tumor-free principle should be strictly followed during surgery (31). For immature teratomas, a standard bleomycin, etoposide, and platinum (BEP) chemotherapy regimen should be established postoperatively. For malignant teratoma, surgery should be the main treatment, supplemented by chemotherapy and radiotherapy. In this case, the tumor was completely resected, and the postoperative pathology was cystic and mature teratoma, which was a benign tumor.

## Data availability statement

The original contributions presented in this study are included in the article/supplementary material, further inquiries can be directed to the corresponding authors.

## Ethics statement

Written informed consent was obtained from the individual(s) for the publication of any potentially identifiable images or data included in this article.

## Author contributions

YY and MZ: acquisition of data and writing—original draft. MC, HT, and ZQ: analysis and interpretation of data and visualization. JL, HW, and MB: perform the analysis with constructive discussions. JC: conceptualization, funding acquisition, resources, supervision, and writing. BX: conceptualization, supervision, and writing. All authors contributed to the article and approved the submitted version.
